# Anti-Metastasis Fascin Inhibitors Decrease the Growth of Specific Subtypes of Cancers

**DOI:** 10.3390/cancers12082287

**Published:** 2020-08-14

**Authors:** Yufeng Wang, J. Jillian Zhang, Xin-Yun Huang

**Affiliations:** 1 Department of Physiology and Biophysics, Weill Cornell Medical College of Cornell University, New York, NY 10065, USA; yuw2007@med.cornell.edu; 2 Novita Pharmaceuticals, Inc., New York, NY 10065, USA; jzhang1161@gmail.com; 3 Sandra and Edward Meyer Cancer Center, Weill Cornell Medical College of Cornell University, New York, NY 10065, USA

**Keywords:** fascin, tumor metastasis, cytoskeleton

## Abstract

Fascin is an actin-bundling protein that is critical for filopodial formation and other cellular cytoskeletal structures. An elevated expression of fascin has been observed in tumor cells and is correlated with a shorter survival of cancer patients. Given its roles in tumor cell migration and invasion, we have developed small-molecule fascin inhibitors to prevent and delay tumor metastasis. Here we report the characterization of a new fascin inhibitor in mice. In addition to its inhibitory effects on tumor metastasis, we also report that fascin inhibitors can decrease the growth of specific subtypes of cancers, including epidermal growth factor receptor (EGFR)-high triple-negative breast cancer, and activated B-cell subtypes of diffuse large B-cell lymphoma. Hence, fascin inhibitors can be used to not only inhibit tumor metastasis, but also decrease the tumor growth of specific cancer types.

## 1. Introduction

Fascin is the main actin cross-linker in filopodia and shows no amino acid sequence homology with other actin-binding proteins [1,2,3,4,5]. Studies using cancer patient biopsies demonstrate that fascin is a biomarker of metastases [6,7,8,9,10]. Elevated fascin mRNA and/or protein levels are found in almost all types of metastatic tumors, and are correlated with clinically aggressive phenotypes, a poor prognosis, and shorter survival [11,12]. Human fascin expression is low or absent in normal adult epithelial cells, but highly expressed in metastatic tumors [13,14]. Fascin gene-knockout mice were normal, likely due to the functional compensation of other actin-bundling proteins during embryonic development [15]. A deletion of the fascin gene delayed tumor development, slowed tumor growth, reduced metastatic colonization, and increased the overall survival in a spontaneous mouse model of pancreatic cancer [16]. Conversely, the transgenic expression of fascin in mouse intestinal epithelium increased the tumor incidence, promoted tumor progression, and decreased the overall survival in a spontaneous mouse model of colorectal cancer [17]. These mouse genetic studies provide strong evidence for fascin in tumor initiation (tumor burden), tumor progression, tumor metastasis and overall survival.

We previously screened chemical libraries and identified small-molecule compounds that specifically inhibit the biochemical function of fascin to bundle actin filaments [18]. One of the initial fascin inhibitor hits was optimized through medicinal chemistry, and the improved fascin inhibitors blocked the actin-binding and actin-bundling activities of fascin, as well as tumor cell migration, invasion and metastasis [19]. To understand the mechanism of action by which fascin inhibitors interfere with the biological functions of fascin, we solved the X-ray crystal structure of fascin and a fascin inhibitor, and revealed that the fascin inhibitor occupies one actin-binding site and induces a large conformational change of fascin to impair the actin-bundling function of fascin [20]. Here we further characterize a new fascin inhibitor, NP-G2-044, in mice. We have shown that NP-G2-044 has a good pharmacokinetic profile in mice, delays the metastatic relapse, and increases the overall survival of tumor-bearing mice. Furthermore, we reveal that NP-G2-044 decreases the cell proliferation and primary tumor growth of activated B-cell diffuse large B-cell lymphoma, diffuse mixed lineage lymphoma, and epidermal growth factor receptor (EGFR)-high triple-negative breast cancer.

## 2. Results

### 2.1. Pharmacokinetic Studies of a Fascin Inhibitor in Mice

**Figure 1:**
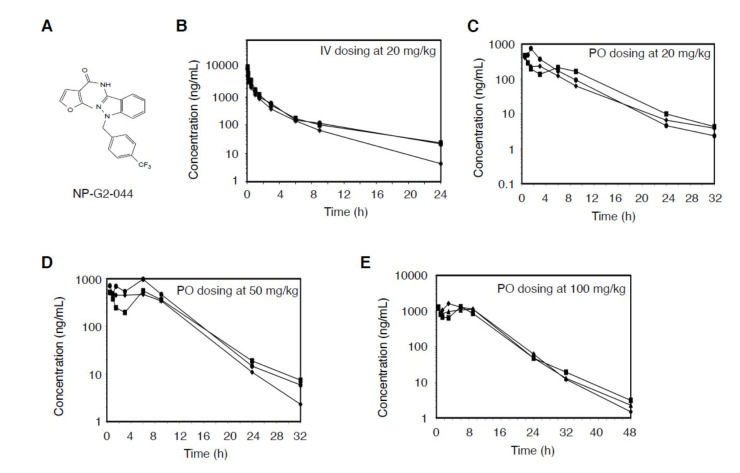
Pharmacokinetic (PK) studies of the fascin inhibitor NP-G2-044 in mice. (**A**) The chemical structure of NP-G2-044. (**B**–**E**) PK profiles of NP-G2-044 in mice. NP-G2-044 was intravenously (IV) (at 20 mg/kg, **B**) or orally (PO) (at 20 mg/kg, **C**; at 50 mg/kg, **D**; or at 100 mg/kg, **E**) administered into mice. Blood samples were collected at different time points. The plasma samples were then extracted and the concentrations of NP-G2-044 were determined by LC-MS/MS. The concentration–time curves are shown. Each curve was from the data of one mouse. Each dose was with three mice.

To facilitate efficacy studies of fascin inhibitors in animals, we first carried out pharmacokinetic (PK) studies of a fascin inhibitor, NP-G2-044, in mice (Figure 1A). The PK studies showed that NP-G2-044 had an excellent PK profile (Figure 1B–E and Table 1). The PK parameters of NP-G2-044 were assessed following a single intravenous (IV) and single oral (PO) gavage dose administration to mice. Blood samples were collected pre-dose, and 3, 10, 30 min, 1, 1.5, 3, 6, 9, 24, 32, or 48 h after dosing. The plasma samples were then extracted and the concentrations of NP-G2-044 were determined by LC-MS/MS. The concentration–time curves are shown in Figure 1B–E. These data were analyzed and the PK parameters for NP-G2-044 are summarized in Table 1. T_max_ ranged from 0.8 to 6 h after oral dosing of 20 mg/kg to 100 mg/kg of NP-G2-044. The half-life (t_½_) was –4 h. The oral bioavailability (based on AUC_0–t_) was 34–39%. After an IV administration in mice, the steady-state volume of distribution (V) of NP-G2-044 was 9.3 L/kg (Table 1), which is much greater than the plasma volume (0.05 L/kg) and the total body water (0.725 L/kg) in mice, suggesting the distribution of NP-G2-004 into tissues [21]. The total plasma clearance (CL) was 48 mL/min/kg (Table 1), which is about half of the hepatic blood flow (90 mL/min/kg) and is greater than the glomerular filtration rate (14 mL/min/kg) in mice [21]. Together, these data demonstrate that NP-G2-044 has excellent PK properties in mice.

### 2.2. Dose-Response Studies of Fascin Inhibitors in Blocking Tumor Metastasis

**Figure 2:**
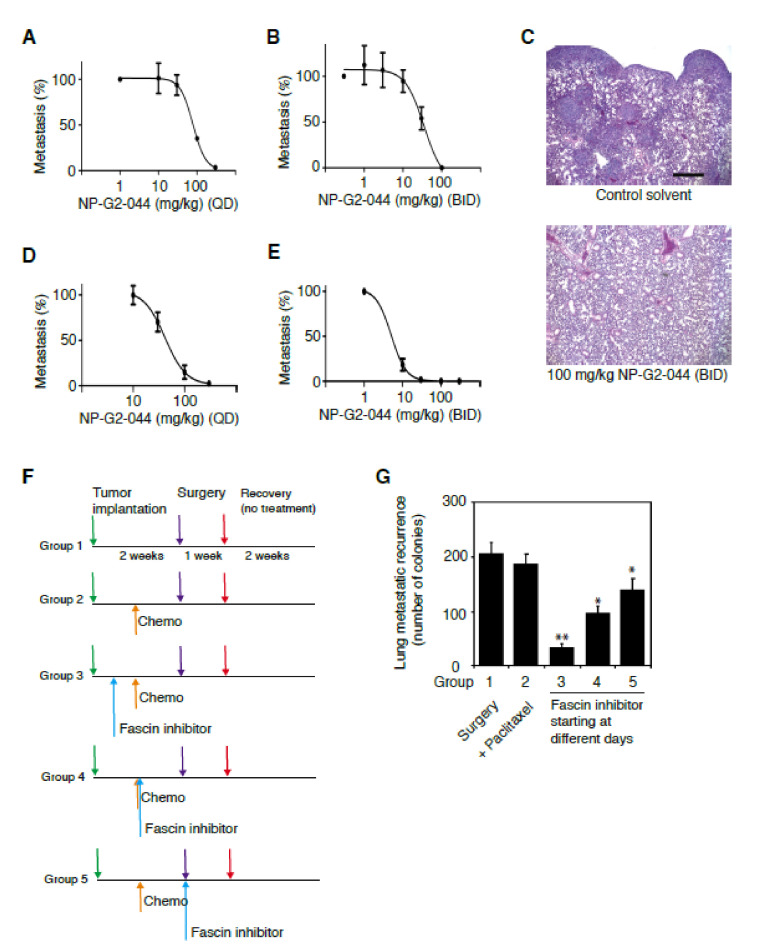
Dose-response studies of fascin inhibitors blocking tumor metastasis in mouse models. (**A**,**B**) MDA-MB-231 human breast tumor cells were implanted into the mammary fat pad and the metastasis to the lung was quantified. QD: once a day. BID: twice a day treatment with different concentrations of NP-G2-044. Each group had 3 to 4 mice. Data are shown as the mean ± SEM. (**C**) Representative images of hematoxylin and eosin staining show lung tissue sections from mice injected with MDA-MB-231 cells treated with a control solvent or treated with 100 mg/kg NP-G2-044. (**D**,**E**) 4T1 mouse breast tumor cells were implanted into the mammary fat pad and the metastasis to the lung was quantified. Each group had 3 to 4 mice. Data are shown as the mean ± SEM. (**F**,**G**) Effect of NP-G2-044 on tumor metastatic recurrence. 4T1 breast tumor cells were implanted into the fat pad. Chemotherapy with paclitaxel (20 mg/kg, twice weekly) was given on Day 7. Primary tumors were surgically removed on Day 14. The metastatic recurrence in the lung was quantified on Day 32. A total of 100 mg/kg of NP-G2-044 was given once daily to mice starting on Day 3 (Group 3), 7 (Group 4) or 14 (Group 5). Each group had 3 mice. Data are shown as the mean ± SD. Student *t*-test: * *p* < 0.05; ** *p* < 0.005. Scale Bar: 100 μm.

Based on the above PK profile (such as t_½_ of –4 h), we next performed the dose-regimen studies of NP-G2-044 on tumor metastasis given once daily or twice a day (Figure 2A–E). We used two mouse models. In the first orthotopic spontaneous tumor metastasis mouse model, MDA-MB-231 human triple-negative breast cancer cells were injected into the orthotopic site (the mammary gland) of immuno-deficient NSG mice, and then the metastasis to the lung was monitored [19,22,23,24] (Figure 2A–C). Seven days after the implantation of MDA-MB-231 tumor cells, we orally administered the mice with different concentrations of NP-G2-044 either once a day or twice a day for six days a week. After eight weeks, the mice were sacrificed and examined for metastasis in the lungs. Comparing with the mice that were given the control solvent, the number of metastasized tumor cells in the lungs of mice treated with NP-G2-044 was significantly reduced. The IC_50_ was 78.7 mg/kg when given once a day, and an almost complete inhibition was seen with 300 mg/kg (Figure 2A). When orally given twice a day, the IC_50_ was 29.2 mg/kg, and an almost completely inhibition was seen with 100 mg/kg (Figure 2B). Lung tumor metastasis was further verified by a histological analysis of lung sections and stained with hematoxylin and eosin (Figure 2C).

In the second orthotopic spontaneous tumor metastasis mouse model, 4T1 mouse triple-negative breast cancer cells were injected into the mammary gland of BALB/c mice, and then the metastasis to the lung was monitored [19,22,23,24] (Figure 2D,E). The 4T1 mouse tumor closely mimics human breast cancer in its anatomical site, immunogenicity, growth characteristics, and metastatic properties [25]. From the mammary gland, the 4T1 tumor spontaneously metastasizes to a variety of target organs including lung, bone, brain, and liver. Seven days after the implantation of 4T1 tumor cells, we orally administered the mice with different concentrations of NP-G2-044 either once a day or twice a day for six days a week. After 28 days, the mice were sacrificed and examined for metastasis in the lungs. Whereas mice given with control solvent exhibited large numbers of metastasized 4T1 cells in the lungs, the number of metastasized 4T1 cells in the lungs of mice treated with NP-G2-044 was markedly reduced. When orally given once a day, the IC_50_ was 40 mg/kg, and an almost complete inhibition was seen with 300 mg/kg (Figure 2D). When orally given twice a day, the IC_50_ was 5 mg/kg, and an almost complete inhibition was seen with 30 mg/kg (Figure 2E). The above data indicate that the total drug exposure over time is more critical than the peak (maximum) serum concentration of NP-G2-044 for the blocking effect on tumor metastasis.

### 2.3. Fascin Inhibitor Slows Breast Cancer Metastatic Relapse

After the surgical removal of primary tumors followed by radiation therapy or chemotherapy, the tumor often comes back in patients with triple-negative breast cancer. To test whether fascin inhibitors could be used as a maintenance therapy to slow the process of tumor relapse, we administered NP-G2-044 after the surgical removal of primary tumors and systematic chemotherapy, and then examined the metastatic recurrence of breast tumors. We implanted 4T1 tumor cells into the mammary glands of mice. The mice were treated with chemotherapy (paclitaxel) starting on Day 7 and the primary tumors were surgically removed on Day 14 (when the primary tumors were visible and could be surgically removed). Starting on Day 3 (Group 3 in Figure 2F,G), 7 (Group 4 in Figure 2F,G), or 14 (Group 5 in Figure 2F,G), NP-G2-044 was given once a day by gavage (Figure 2F). All treatments were stopped at Day 21, and the mice were sacrificed on Day 32 for the examination of lung metastatic recurrence (Figure 2F,G). The data showed that treatments with NP-G2-044 slowed the metastatic recurrence of breast tumors in the lung. For example, compared to chemotherapy alone, treatment with NP-G2-044 (such as starting on Day 3) slowed the metastatic recurrence (*p* < 0.05), and the earlier the treatment the better the efficacy (Figure 2G). These data suggest that it might be possible to use NP-G2-044 as a maintenance therapy to decrease the metastatic recurrence right after the surgical removal of primary tumors and chemotherapy in patients.

### 2.4. Fascin Inhibitor Alone and in Combination With Chemotherapy Increase the Overall Survival of Tumor-Beaing Mice

**Figure 3:**
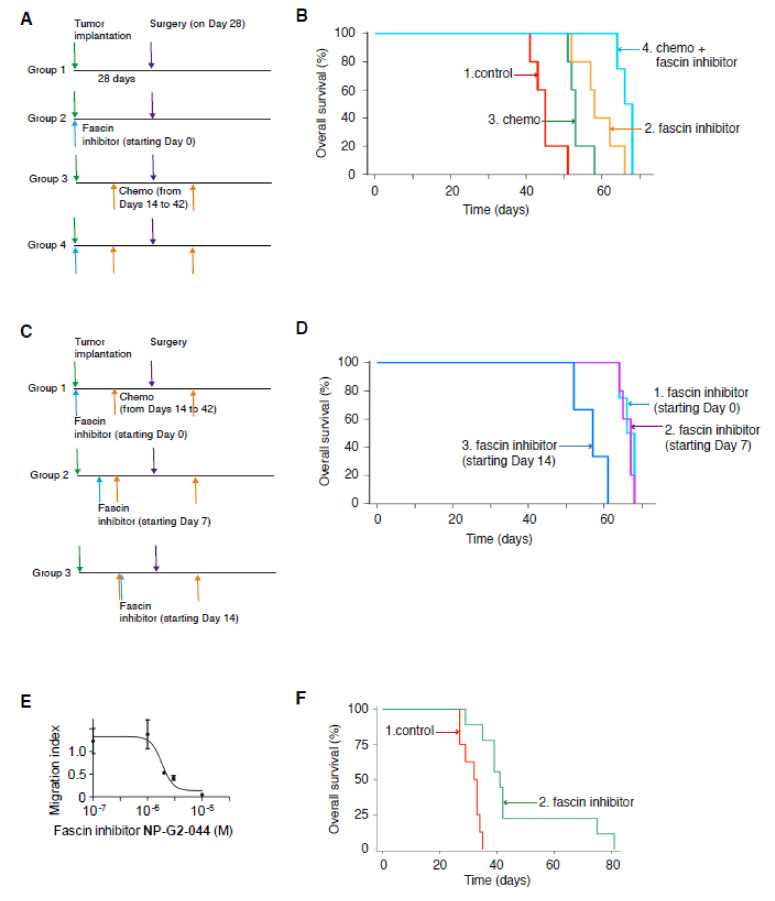
Fascin inhibitor NP-G2-044 increases the overall survival of tumor-bearing mice. NSG mice implanted with MDA-MB-231 tumor cells were treated with a fascin inhibitor, chemotherapy, or a combination of a fascin inhibitor + chemotherapy. Primary tumors were surgically removed on Day 28. Chemotherapy treatment was for 4 weeks (as marked). NP-G2-044 started on Day 0. (**A**,**B**) The fascin inhibitor, chemotherapy and the combination all increased the overall survival of tumor-bearing mice. (**A**) Experimental schemes for the data are shown in (**B**). (**B**) The overall survival curves of mice from the four groups of mice. (**C**,**D**) In the combination therapies, earlier treatments with NP-G2-044 (starting on Day 0 or 7) had a better effect than late treatment (starting on day 14). (**C**) Experimental schemes for the data shown in (**D**). (**D**) The overall survival curves of mice from the three different groups. The group with starting Day 0 was the same one as the fourth group in (**B**). Death was used as the endpoint. (**E**) Inhibition of the migration of NCI-H660 human neuroendocrine prostate cancer cells by NP-G2-044 in the presence of serum. (**F**) NP-G2-044 increases the overall survival of mice bearing NCI-H660 cancer cells. NCI-H660 human neuroendocrine prostate cancer cells were subcutaneously injected into the prostate of male NSG mice. In the control group, the vehicle solvent was given (marked as “control”). In the second group, the mice were treated with NP-G2-044. Death was used as the endpoint.

Successful regulatory drug approval usually hinges on an overall survival benefit. Therefore, we examined the effect of NP-G2-044 on the overall survival of tumor-bearing mice. Moreover, for breast tumor cells, NP-G2-044 did not induce the apoptosis of 4T1 or MDA-MB-231 triple-negative breast cancer cells [18,19]. Therefore, it is possible that anti-migration agents such as the fascin inhibitor, when combined with cytotoxic agents such as cyclophosphamide and doxorubicin, will lead to an even greater benefit. Therefore, we also investigated the combination therapy of fascin inhibitors with chemotherapy. In these experiments, MDA-MB-231 human triple-negative breast cancer cells were implanted into the mammary gland of immuno-deficient NSG mice. In one group, control solvents were given. In the second group, the mice were treated with NP-G2-044. The third group of mice was treated with chemotherapy (cyclophosphamide + doxorubicin). The fourth group of mice was treated with NP-G2-044 combined with chemotherapy (Figure 3A). The primary tumors were surgically removed on day 28 (Figure 3A). Compared with the control group, NP-G2-044 treatment (once a day for 6 days/week) increased the median overall survival by ~29% (log-rank *p* = 0.002) (Figure 3B). Chemotherapy alone (cyclophosphamide + doxorubicin, once every week) increased the median overall survival by ~18% (log-rank *p* = 0.005) (Figure 3B). A combination of NP-G2-044 and chemotherapy increased the median overall survival by ~50% (log-rank *p* = 0.005) (Figure 3B). Furthermore, we examined the effect of treatments with fascin inhibitors starting at different time points (Figure 3C,D). Starting NP-G2-044 treatments on Day 0 and Day 7 led to similar effects on the overall survival (Figure 3D). These earlier treatments with NP-G2-044 (starting on Day 0 and Day 7) had a longer overall survival than the treatment starting on Day 14 (Figure 3D). These studies indicate that anti-migration agents can extend the life of tumor-bearing mice.

To investigate whether this increased effect on the overall survival might be limited to triple-negative breast cancers, we used NCI-H660 human neuroendocrine prostate cancer cells as an additional model (Figure 3E,F). First, we tested the effect of NP-G2-044 on the migration of NCI-H660 cells (Figure 3E). We used the quantitative Boyden chamber assay. NCI-H660 tumor cells were loaded onto the top of the Boyden chamber. After about twenty hours, the cells migrated into the bottom of the chamber filter and were counted. NP-G2-044 blocked NCI-H660 tumor cell migration with an IC_50_ value of 3–5 μM (Figure 3E). Since a plasma protein binding assay with mouse plasma showed a fraction unbound value of ~0.3% (in the presence of 100% serum) for NP-G2-044, the actual IC_50_ value for free NP-G2-044 is 90–150 nM (in the presence of 10% serum), which is similar to the IC_50_ value obtained from the in vitro actin-bundling assay [19]. To investigate the effect of NP-G2-044 on the overall survival of mice bearing NCI-H660 tumors, NCI-H660 tumor cells were injected into the prostate of male NSG mice. NCI-H660 tumor-bearing mice were randomized into two groups. In the control group, the vehicle solvent was given (marked as the “control” group) (Figure 3F). In the second group, the mice were treated with NP-G2-044 (5 days per week by an oral gavage). NP-G2-044 treatment started on Day 7. Comparing with the control group, NP-G2-044 treatment increased the median overall survival by 50% (log-rank *p* < 0.001) (Figure 3F). These data again demonstrate a beneficial effect on the overall survival by NP-G2-044.

### 2.5. Inhibition of the Tumor Growth of the Activated B-cell (ABC) Subtype of Diffuse Large B-cell Lymphoma (DLBCL) and Diffuse Mixed Lymphoma by NP-G2-044

**Figure 4:**
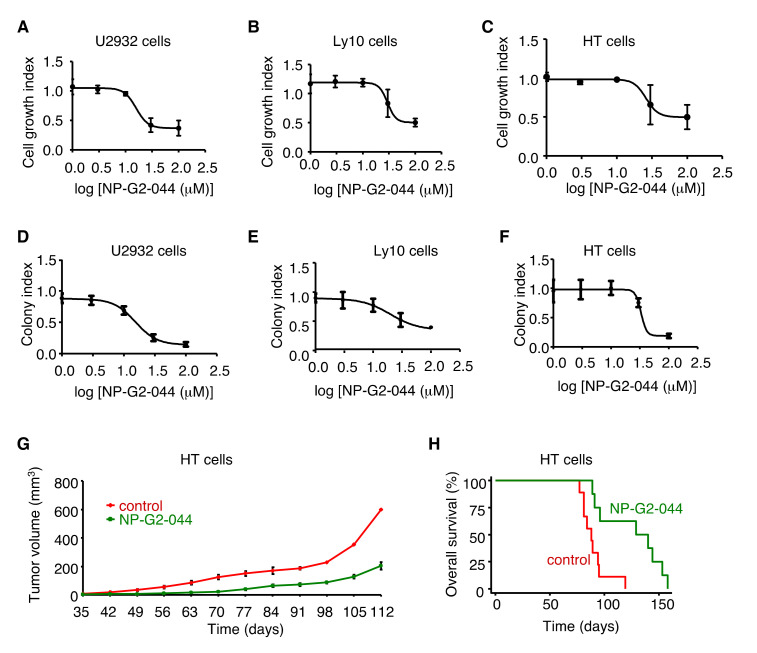
Growth inhibition of the activated B-cell (ABC)-subtype of diffuse large B-cell lymphoma (DLBCL) and diffuse mixed lymphomas by NP-G2-044. (**A**–**C**) NP-G2-044 suppresses the cell growth of three NP-G2-044 sensitive lymphoma cell lines. U2932, Ly10, and HT cells were growing in the presence of different concentrations of NP-G2-044. The cell numbers were counted after 4 days. Data are the mean ± SD. *n* = 3. (**D**–**F**) Effect of NP-G2-044 on soft agar colony formation of three different lymphoma cell lines. U2932, Ly10, and HT cells were growing in soft agar in the presence of different concentrations of NP-G2-044. The colony numbers were counted after 14 days. Data are the mean ± SD. *n* = 3. (**G**) NP-G2-044 decreases HT lymphoma growth. HT lymphoma cells were subcutaneously injected into NSG mice, and divided into two groups. The tumor volume was measured every week until the mice died. The NP-G2-044-treated group decreased the tumor growth compared with the control group (*p* < 0.05). Data are shown as the mean ± SD (*n* = 9 or 8). (**H**) Overall survival curves of HT lymphoma-bearing mice from the control group, and the NP-G2-044-treated group. Logrank test, *p* = 0.002.

**Figure 5:**
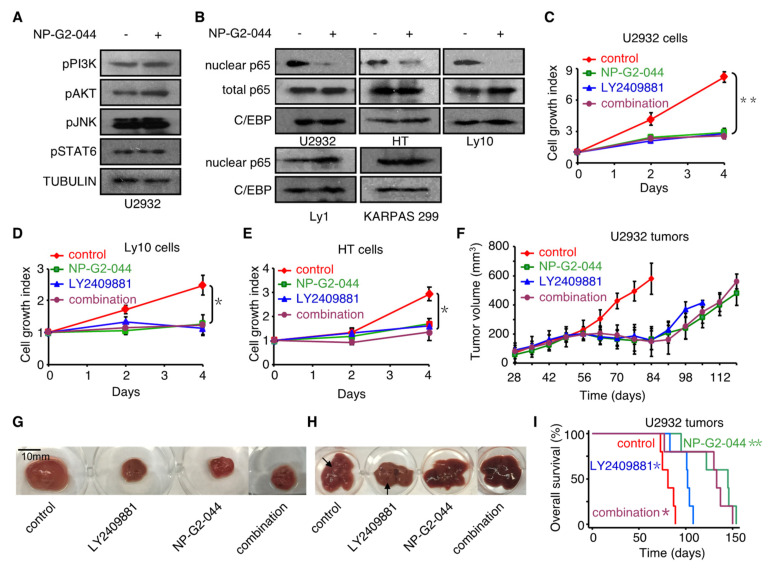
Inhibition of signaling pathways in lymphoma cells by NP-G2-044. (**A**) Effect of NP-G2-044 on the PI3K, AKT, JNK and STAT6 signaling pathways in DLBCL lymphoma cells. Phosphorylation of PI3K, AKT, JNK, and STAT6 was detected by Western blotting. Tubulin was used as control. Whole-cell lysates of U2932 cells were used. (**B**) NP-G2-044 suppresses NF-κB nuclear translocation in three lymphoma cell lines. Nuclear extracts and whole-cell lysates were used to check the protein levels of p65 in cells treated with or without NP-G2-044. C/EBP, a nuclear protein, was used as control. The top three cell lines, but not the bottom two cell lines, were sensitive to NP-G2-044. (**C**–**E**) NP-G2-044 suppresses cell growth of three NP-G2-044 sensitive lymphoma cell lines. U2932, Ly10, and HT cells were growing in the absence (control) or presence of NP-G2-044, the NF-κB inhibitor LY2409881, or the combination of NP-G2-044 and LY2409881 (marked as “combination”). The cell numbers were counted every other day for 4 days. * *p* < 0.05. (**F**) NP-G2-044 decreases U2932 lymphoma growth. U2932 lymphoma cells were subcutaneously injected into NSG mice, and divided into four groups. The tumor volume was measured every week until the mice died. The treatment groups (NP-G2-044, LY2409881, and the combination of NP-G2-044 + LY2409881) all decreased the tumor growth to a similar degree, compared to the control group. (**G**) Examples of primary tumor tissues from U2932 lymphoma cell-implanted mice from the control group and the treatment groups (NP-G2-044, LY2409881, and the combination of NP-G2-044 + LY2409881). (**H**) Examples of liver metastasis from U2932 lymphoma cell implanted mice from the control group and the treatment groups (NP-G2-044, LY2409881, and the combination of NP-G2-044 + LY2409881). (**I**) Overall survival curves of U2932 lymphoma-bearing mice from the control group, and the treatment groups (NP-G2-044, LY2409881, and the combination of NP-G2-044 + LY2409881). Logrank test, * *p* < 0.02; ** *p* < 0.002.

To search for subtypes of cancers with tumor cell growth sensitive to NP-G2-044, we started from lymphoma since fascin has been used for the diagnosis of classical Hodgkin lymphoma for more than 20 years (100% of Reed-Sternberg cells express fascin proteins) [26]. We tested different concentrations of NP-G2-044 on the cell growth of various classical Hodgkin lymphoma cells including L-1236, L428, KM-H2, HDLM-2, U-HO1, and SUP-HD1 cells, and did not observe an inhibitory effect of NP-G2-044 on the cell growth of these lymphoma cells in culture. We then extended our search to other types of lymphoma cells. From 12 different types of lymphoma cells, we observed an inhibitory effect of NP-G2-044 on the cell growth of U2932, Ly10, and HT cells (Figure 4A–C). Both U2932 and Ly10 cells are ABC-subtype DLBCLs, and HT cells are diffuse mixed lymphoma. To confirm these inhibitory effects on cell growth, we performed soft-agar colony formation assays. NP-G2-044 decreased the number of growing colonies of these cells in a dosage-dependent manner (Figure 4D–F), reaffirming the inhibitory effect of NP-G2-044 on the proliferation of these lymphoma cells. To assess the effect on the primary tumor growth in animal models, we injected U2932 cells or HT cells into the abdomen of NSG mice. These mice were then treated with a control solvent or NP-G2-044. In both cases, the primary tumor growth was decreased, and the overall survival was increased by NP-G2-044 treatment (Figure 4G,H and see below Figure 5F,I).

To understand the molecular mechanism of action by which NP-G2-044 decreases the growth of U2932 and HT cells, we examined the effects of NP-G2-044 on several cellular signaling pathways in these cells (Figure 5A,B and Figures S1 and S2). Previous studies showed that the growth of the ABC-subtype of DLBCL cells depends on the NF-κB pathway and the PI3K pathway [27]. We treated U2932 cells with or without NP-G2-044, and measured the potential effects of NP-G2-044 on various pathways critical for lymphoma cell growth. While NP-G2-044 had no effect on the phosphorylation of PI3K, AKT kinase, JNK kinase, and STAT6 transcription factor, it decreased the nuclear translocation of the p65 subunits (a subunit of NF-κB) (Figure 5A,B). These effects on p65 nuclear translocation were also observed in Ly10 and HT cells, but not in the NP-G2-044 insensitive Ly1 and KARPAS 299 lymphoma cells (Figure 5B). Furthermore, we confirmed the dependence of the growth of U2932, Ly10 and HT cells on the NF-κB pathway by showing that the growth of these cells was sensitive to LY2409881, a small-molecule inhibitor of IkB kinase which is critical for the activation and nuclear translocation of p65 [28] (Figure 5C–E).

Moreover, the inhibition of NF-κB signaling by the IkB kinase inhibitor LY2409881 decreased the tumor growth in mice injected with U2932 cells (Figure 5F,G). This decrease was similar to those observed with NP-G2-044, as well as for the combination of NP-G2-044 + LY2409881 (Figure 5F,G). We also studied the effect on the metastasis of the implanted lymphoma cells into the liver (Figure 5H). While NP-G2-044 and the combination of NP-G2-044 + LY2409881 blocked the metastasis to the liver, LY2409881 alone did not (Figure 5H). Additionally, NP-G2-044 increased the median overall survival by 79% (log-rank, *p* = 0.0018), and LY2409881 increased the median overall survival by 26% (log-rank, *p* = 0.013) (Figure 5I). The less dramatic effect of LY2409881 on the overall survival might be due to the metastasis observed in the LY2409881-treated mice (Figure 5H). The combination treatment (NP-G2-044 + LY2409881) had a similar effect as NP-G2-044 (Figure 5I). Together, the above data demonstrate that NP-G2-044 decreased the growth of U2932 and HT lymphoma cells in culture and in animal models.

### 2.6. Inhibition of Tumor Growth of EGFR-High Triple-Negative Breast Cancers by NP-G2-044

**Figure 6:**
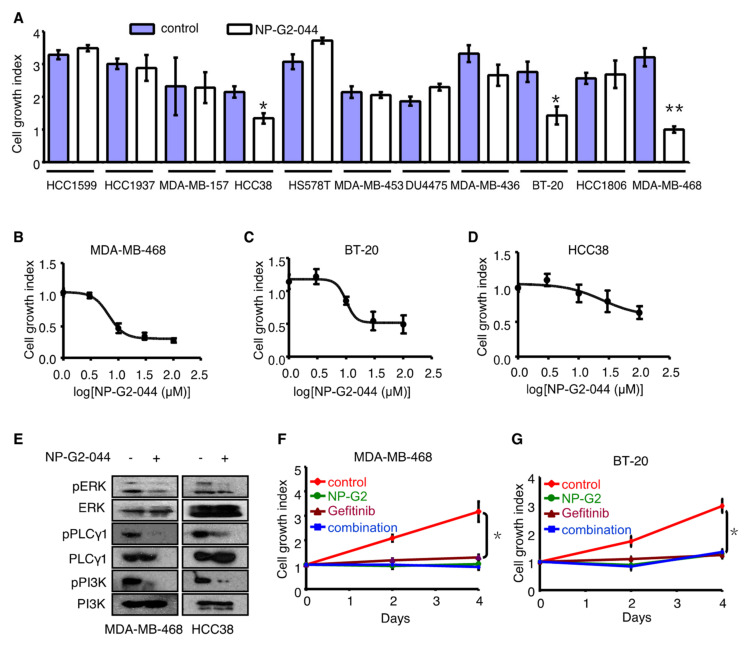

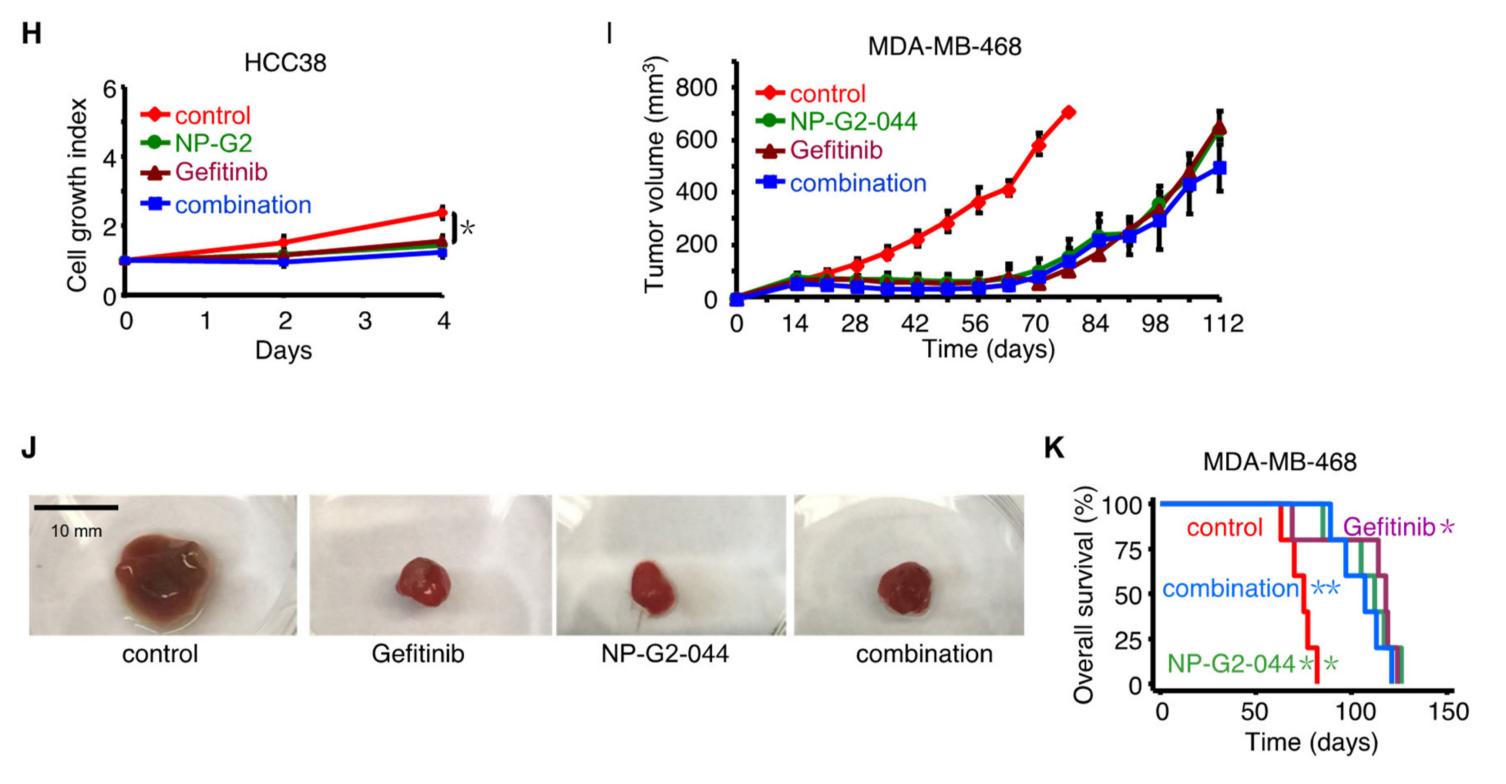
NP-G2-044 inhibition of tumor growth of epidermal growth factor receptor (EGFR)-high triple-negative breast cancer (TNBC) tumors in mouse models. (**A**) Effect of NP-G2-044 on the cell growth of 11 TNBC cell lines. Cells were growing in the presence of 10% serum with or without NP-G2-044. The number of cells was counted after 4 days of culture. (**B**–**D**) Dose-dependent inhibition of cell growth of three fascin inhibitor-sensitive TNBC cell lines by NP-G2-044. Data are shown as the mean ± SD (*n* = 3). Student t-test, * *p* < 0.05; ** *p* < 0.005. (**E**) NP-G2-044 suppresses the EGFR signaling pathway. Phosphorylation of ERK, PLCγ1, and PI3K was detected by Western blotting. The un-phosphorylated forms were used as the control. Whole-cell lysates of MDA-MB-468 and HCC38 cells were used. (**F**–**H**) NP-G2-044 suppresses the cell growth of three NP-G2-044 sensitive TNBC cell lines. MDA-MB-468, BT-20, and HCC38 cells were growing in the absence (control) or presence of NP-G2-044, the EGFR inhibitor gefitinib, or the combination of NP-G2-044 and gefitinib (marked as “combination”). The cell numbers were counted every other day for 4 days. * *p* < 0.05. (**I**) NP-G2-044 decreases MDA-MB-468 tumor growth. MDA-MB-468 tumor cells were implanted into the fat pat, and divided into four groups. The tumor volume was measured every week until the mice died. The treatment groups (NP-G2-044, gefitinib, and the combination of NP-G2-044 + gefitinib) all decreased the tumor growth to a similar degree, comparing with the control group. (**J**) Examples of primary tumor tissues from MDA-MB-468 tumor cell implanted mice from the control group and the treatment groups (NP-G2-044, gefitinib, and the combination of NP-G2-044 + gefitinib). (**K**) Overall survival curves of MDA-MB-468 tumor-bearing mice from the control group, and the treatment groups (NP-G2-044, gefitinib, and the combination of NP-G2-044 + gefitinib). Logrank test, * *p* < 0.05; ** *p* < 0.002.

In our previous studies with triple-negative breast cancer (TNBC) cells, such as MDA-MD-231, EMT-6, and 4T1 cells, we did not see an inhibitory effect of fascin inhibitors on the growth of these cells in culture and the growth of primary tumors in animal models [18,19]. During our testing of various TNBC cells for their growth sensitivity to fascin inhibitors, we noticed that the growth of MDA-MB-468, BT-20, and HCC38 triple-negative breast cancer cells were sensitive to fascin inhibitors (Figure 6A–D). Among these three cell lines, MDA-MB-468 cells were the most sensitive (Figure 6A). We searched various molecular classifications of TNBCs, and noticed that these three cell lines belong to the same cluster [29]. The EGFR amplification was exclusive to this cluster of TNBC cell lines [29]. MDA-MB-468 cells have the highest expression of EGFR proteins among all TNBC cells, with BT20 cells being the second [29]. Both cell lines have more than seven copies of the EGFR gene [29]. Indeed, the growth of these cells was sensitive to the EGFR inhibitor gefitinib as well as NP-G2-044 (Figure 6F–H).

To understand the molecular mechanism of action by which NP-G2-044 decreases the growth of EGFR-high triple-negative breast cancer cells, we investigated the effect of NP-G2-044 on the signaling steps downstream of EGFR (Figure 6E and Figure S3). MDA-MB-468 cells and HCC38 cells grew in the presence of EGF with or without NP-G2-044. The activation of multiple signaling pathways were examined, and we found that the phosphorylation of ERK, PLCγ1, and PI3K were all affected by NP-G2-044 treatment (Figure 6E). This would imply that NP-G2-044 might act on an early step of EGFR signaling, or even on EGFR itself since EGFR has been shown to directly interact with the actin cytoskeleton [30,31,32]. Finally, we used the animal model to investigate whether the primary tumor growth of MDA-MB-468 cells was sensitive to fascin inhibitors. MDA-MB-468 tumor cells were implanted into the mammary gland of NSG mice, and these mice were randomly divided into four groups. One group of mice was used as control (Figure 6I). The second group of mice was treated with the fascin inhibitor NP-G2-044 (Figure 6I). The third group was treated with the EGFR inhibitor gefitnib (Figure 6I), and the fourth group was treated with the combination of NP-G2-044 + gefitinib (Figure 6I). Comparing with the control group, the NP-G2-044 treatment, gefitinib treatment, and the combination treatment reduced the growth of primary tumors to the same degree (Figure 6I). The reduction in tumor mass was confirmed by the smaller sizes of isolated tumor tissues from the treated mice (Figure 6J). We dissected the primary tumor tissues 55 days after the tumor implantation from some mice. Tumor tissues from the control group had bigger tumor masses than those from the other three treatment groups (Figure 6J). Furthermore, the overall survival of the mice was analyzed (Figure 6K). Compared with the control group, the three treatment groups increased the median overall survival by ~50%. We did not observe an additive or synergistic effect by NP-G2-044 and gefitinib on the primary tumor growth or on the overall survival (Figure 6I,K). The simplest interpretation is that fascin and EGFR act on the same signaling pathway, and thus the inhibition of both fascin and EGFR would block the same pathway leading to similar effects. Together, the above data demonstrate that NP-G2-044 inhibits the tumor growth of MDA-MB-468 cells in culture and in animal models.

## 3. Discussion

Tumor metastasis is responsible for ~90% of death from cancers, and the prevention and delay of tumor metastasis will significantly increase the survival of cancer patients [33,34,35]. Tumor cell migration and invasion are essential for metastasis [36]. Cell migration requires actin cytoskeleton reorganization to cause dynamic changes in cell shapes [37]. One of the most prominent plasma membrane protrusions is filopodia [38]. Metastatic tumor cells are rich in filopodia, and the numbers of filopodia correlate with their invasiveness [38,39,40]. We have shown that the fascin inhibitor NP-G2-044 has an excellent PK profile in mice. Both once-a-day and twice-a-day dosing regimens could lead to the complete inhibition of tumor metastasis in animal models. It took lower doses to reach a similar effect when given twice-a-day, indicating that the drug exposure, rather than the peak drug concentration, is critical for the drug anti-metastasis efficacy. Our data suggest that fascin inhibitors might slow tumor recurrence after primary tumor removal. Therefore, it is possible to use fascin inhibitors as a maintenance therapy to prolong cancer disease control. Furthermore, we have shown that NP-G2-044 decreases the growth of U2932 and Ly10 cells (both are ABC-subtype of DLBCL cells) and HT cells (a diffuse mixed lymphoma cell), as well as MDA-MB-468 and BT-20 cells (both are EGFR-high triple-negative breast cancer cells). Although in some experiments the number of mice used per group was relatively small, there were different doses of NP-G2-044 used and different types of experiments, and the data were consistent. All the cell lines used in this study express fascin proteins, as confirmed by Western blots with anti-fascin antibodies.

We have shown that NP-G2-044 can prolong the overall survival of mice bearing triple-negative breast tumors, neuroendocrine prostate cancer, or certain subtypes of lymphomas. Triple-negative breast cancer is molecular subtype of breast cancer that is difficult to treat. Triple-negative breast cancer patients have the worst prognosis with the majority of patients relapsing within 5 years from diagnosis. In fact, although no more than 15% of breast cancer patients have a triple-negative breast cancer at diagnosis, it can represent 25 to 40% of patients of the total breast cancer population with metastases, accounting for a disproportionately high percentage of the deaths from breast cancer [41]. This is a group of patients that are in desperate need of new strategies to prevent and treat their disease. Similarly, neuroendocrine prostate cancer (NEPC) is a hormone-refractory late manifestation of prostate cancer and represents ~25% of late-stage diseases. NEPC has a poor prognosis, with most patients surviving for less than one year after diagnosis [42,43]. We have shown that fascin inhibitors can inhibit the migration, invasion and metastasis of triple-negative breast cancer cells and neuroendocrine prostate cancer cells, as well as increase the overall survival of mice bearing triple-negative breast tumors or NEPC. Currently, there are no approved drugs on the market that specifically prevent or delay tumor metastasis. One reason is that clinical oncological drug development generally relies on the demonstration of tumor shrinkage. Yet drugs that specifically target the metastasis process might not induce tumor cell death or slow primary tumor growth. There is an urgent need to have anti-metastasis therapeutic agents that also possess inhibitory effects on the growth of certain subtypes of cancers. These anti-metastasis drugs could then be approved first based on their tumor shrinkage efficacy, and their anti-metastasis potential could be clinically demonstrated later. Our data presented here suggest that ABC-subtype of DLBCL, diffuse mixed cell lymphoma, and EGFR-high TNBC can be used as first indications for the future clinical developments of NP-G2-044.

While NP-G2-044 had no effect on the phosphorylation of PI3K, AKT kinase, JNK kinase, and STAT6 transcription factor, it decreased the nuclear translocation of the p65 subunits of NF-κB in ABC-subtypes of DLBCLs (Figure 5A,B). Previously, the overexpression of fascin had been shown to increase the nuclear translocation of NF-κB in MDA-MB-231 cells [44]. This new role of fascin in protein nuclear-cytoplasmic translocation has recently gained intense attention. In addition to its localization in the cytoplasm, fascin has been recently reported to be localized in the nuclear periphery and within the nucleus [45]. In Drosophila, prostaglandins have been shown to regulate the translocation of fascin into and out of the nucleus [45]. Fascin controls nuclear plasticity through a direct interaction with Nesprin-2 that is a component of the LINC (Linker of the Nucleoskeleton and Cytoskeleton) complex known to couple the F-actin cytoskeleton to the nuclear envelope [46]. Depleting fascin or specifically blocking the fascin–Nesprin-2 complex led to defects in nuclear polarization, movement, and cell invasion. Furthermore, fascin is critical for nuclear movement and deformation in migrating cells [46]. Moreover, within the nucleus, fascin regulates the nucleolar structure. It is also possible that fascin inhibitors affect the nuclear actin filament reorganization. Therefore, fascin has newly discovered functions in the nuclear periphery and within the nucleus.

NP-G2-044 treatment decreased the phosphorylation of ERK, PLCγ1, and PI3K downstream of EGF stimulation in EGFR-high triple-negative breast cancer cells (Figure 6E). We previously showed that fascin inhibitors could impair various actin cytoskeletal structures including inhibiting stress fiber formation, focal adhesion turnover, lamellipodial formation, and filopodial formation [19]. The inter-connection of EGFR signaling with the cytoskeleton was observed before [30,31,32]. Recently, the interdependent nature of EGFR and the cytoskeleton have been shown to be critical for various cellular processes, such as cell proliferation and migration [47,48,49,50]. EGFR signaling can induce cytoskeletal reorganization, and the cytoskeleton can regulate EGFR signaling [50]. The cytoskeleton regulates the strength, location, duration, and termination of signaling through a diverse set of mechanisms including force generation and sequestration of signaling regulators [50]. For example, cytoskeletal remodeling could cause rapid EGFR internalization from the plasma membrane, and led to the decrease in its activation of ERK [49]. The cytoskeletal network can transmit mechanical forces that affect EGFR signaling and trafficking at the plasma membrane [47,48,49]. Furthermore, PI3K signaling has also been shown to depend on the actin cytoskeleton [51]. PI3K initiates downstream signaling events to promote cell growth and metabolism. This PI3K signaling allows for the physical dissociation of aldolase from F-actin polymers into the cytoplasm where it is active, thus achieving the coordination of actin cytoskeletal dynamics and glycolysis [51]. A number of glycolytic enzymes have been reported to be associated with the actin cytoskeleton [52,53]. Therefore, components of the cytoskeleton can act as both regulators and targets in cell signaling pathways, integrating cell shape and movement with other cellular responses. Future studies are needed to understand the complex issue of how growth factor signaling coordinates with the cellular cytoskeletal networks to modulate complex cell behaviors. Moreover, although one shared feature of MDA-MB-468 and BT-20 cells is amplified EGFR, there might be other shared features among these cells (as well as HCC38 cells, but not other TNBC cells) that might also contribute to the growth sensitivity of NP-G2-044. HCC38 cells are without EGFR amplification [29]. MDA-MB-468, BT20, and HCC38 cells are all basal-like 1 TNBC cells that are characterized by high mitotic indices and rates of proliferation [29]. EGFR is commonly expressed in these basal-like 1 tumors and promotes cell proliferation via the activation of the Ras/MAPK pathway. MDA-MB-468, BT20 and HCC38 cells are all with TP53 and BRCA2 mutations [29]. Further work will be necessary to explore the possible relationship between these two genes (and their associated signaling pathways such as the cellular response to DNA damage) and the growth sensitivity to fascin inhibitors. In the future, we hope to develop fascin inhibitors as a new treatment to benefit cancer patients.

## 4. Materials and Methods

### 4.1. Mouse Colony

Female BALB/c mice (female 6- to 8-week old) were purchased from Charles River Labs. NSG (NOD.Cg-*Prkdc*^scid^ *Il2rg*^tm1Wjl^/SzJ) immunodeficient mice (female and male 6- to 10-week old) were purchased from the Jackson Laboratory. Studies using mice were performed in compliance with the Institutional Animal Care and Use Committee of Weill Cornell Medical College of Cornell University (Protocol #0709-670A). All mice were housed in the facility of the Research Animal Resource Center of Weill Cornell Medical College of Cornell University.

### 4.2. Cell Culture

The 4T1 mammary tumor cells, human MDA-MB-231 breast cancer cells, and human NCI-H660 neuroendocrine prostate cancer cells were obtained from American Type Culture Collection. The 4T1 cells and MDA-MB-231 cells were cultured in DMEM supplemented with 10% FBS. NCI-H660 were cultured in RPMI-1640 supplemented with 10% FBS. Ly1, Ly10, KARPAS 299, U2932, and HT cells were gifts from Drs. Ethel Cesarman, Ari Melnick and Anas Younes. These cells were cultured in RPMI-1640 supplemented with 10% FBS. HCC1599, HCC1937, MDA-MB-157, HCC38, HS578T, MDA-MB-453, DU4475, MDA-MB-436, BT-20, HCC1806, and MDA-MB-468 (gifts from Dr. Vivek Mittal and Dr. Kristy A. Brown) were cultured in DMEM supplemented with 10% FBS, as previously described [18,19,24].

### 4.3. Cell Growth Assay

For NP-G2-044 dose-dependent studies, 1 × 10^5^ Ly10, U2932, or HT cells were seeded in a 12- or 24-well plate on day 0. A total of 1, 3, 10, 30, or 100 μM NP-G2-044 or control solvent was added to the plates. Cells were collected and counted on day 4. For time-dependent studies, 1 × 10^5^ Ly10, U2932, or HT cells were seeded in a 12- or 24-well plate on day 0. A total of 30 μM NP-G2-044, 5 μM LY2409881, 30 μM NP-G2-044 plus 5 μM LY2409881, or control solvent was added to the plates. Cells were collected and counted on Days 2 and 4. For breast cancer cell growth assays, 1 × 10^5^ HCC1599, HCC1937, MDA-MB-157, HCC38, HS578T, MDA-MB-453, DU4475, MDA-MB-436, BT-20, HCC1806, or MDA-MB-468 cells were seeded in a 12- or 24-well plate on day 0. A total of 30 μM NP-G2-044 or control solvent was added to the plates. Cells were collected and counted on Day 4. For NP-G2-044 dose-dependent studies, 1 × 10^5^ HCC38, BT-20, or MDA-MB-468 cells were seeded in a 12- or 24-well plate on day 0. A total of 1, 3, 10, 30, or 100 μM NP-G2-044, or control solvent was added to the plates. Cells were collected and counted on Day 4. For time-dependent studies, 1 × 10^5^ HCC38, BT-20, or MDA-MB-468 cells were seeded in a 12- or 24-well plate on day 0. A total of 30 μM NP-G2-044, 1 μM gefitinib, 30 μM NP-G2-044 plus 1 μM gefitinib, or control solvent was added to the plates. Cells were collected and counted on Days 2 and 4. The cell growth index was expressed as the fold changes of cell numbers over the starting cell number. For these assays, the old medium was changed with a fresh medium containing the control solvent or the inhibitors every two days.

### 4.4. Colony Formation Assay

A total of 1 × or 2 ×10^4^ cells were suspended in 0.5 mL of 0.3% low melting point agar (Merck KGaA, Darmstadt, Germany) in RPMI-1640 or DMEM containing 10% FBS and control solvent or the inhibitors. This suspension was overlaid on pre-solidified with 0.6% agar in the same medium in 24-well plates as previously described [54]. Normal growth medium containing control solvent or the inhibitors was gently layered over the cultures every 4 days for 14 days. The colonies were stained with 0.1% crystal violet for 1 h at room temperature and counted under a microscope.

### 4.5. Western Blotting

The lymphoma cells were plated in 10-cm dish. After culture for 24 h, cells were incubated in serum-free medium for 16 h. Cells were then incubated in growth medium with 30 µM NP-G2-044 or dissolvent for 16 h, followed by collecting whole cell lysates or nuclear extracts as previously described [55]. The phosphorylation of PI3K, AKT, JNK, and STAT6 was detected using anti-Phospho-PI3K, anti-Phospho-AKT, anti-Phospho-JNK and anti-Phospho-STAT6 antibodies (Santa Cruz Biotechnology, Inc., Dallas, TX, USA, or Cell Signaling Technology, Inc., Danvers, MA, USA). Nuclear p65, total p65, and nuclear C/EBP were detected using anti-p65 and anti-C/EBP antibodies (Santa Cruz Biotechnology, Inc.).

For the cell signal pathway analysis of breast cancer cells, HCC38 and MDA-MB-468 cells were plated in a 10-cm dish. After culture for 24 h, cells were incubated in serum-free medium for 16 h. Cells were then incubated in serum-free medium containing 10 ng/mL EGF with 30 µM NP-G2-044 or dissolvent for 24 h, followed by collecting the whole cell lysate. The phosphorylation of ERK, PLCγ1 and PI3K was detected using anti-Phospho-ERK, anti-Phospho-PLCγ1, and anti-Phospho-PI3K antibodies (Santa Cruz Biotechnology, Inc. or Cell Signaling Technology, Inc.). The total ERK, PLCγ1 or PI3K was detected using anti-ERK, anti-PLCγ1, and anti-PI3K antibodies (Santa Cruz Biotechnology, Inc. or Cell Signaling Technology, Inc.).

### 4.6. Pharmacokinetics Study of NP-G2-044 in Mice

The concentrations of NP-G2-044 in plasma were determined using high performance liquid chromatography with tandem mass spectrometry (LC-MS/MS). All blood samples were transferred into a commercial tube containing potassium (K2) EDTA (ethylene diaminetetraacetic acid) and were processed for plasma. Samples were centrifuged (3000× *g* for 10 min at 2 to 8 °C) within one hour of collection. The assays used a Sciex API 4000 detector and nifedipine as an internal standard. The calibration ranges for NP-G2-044 were from 5.00 to 5000 ng/mL. The plasma concentration of NP-G2-044 in mice was subjected to a non-compartmental pharmacokinetic analysis by using the Phoenix WinNonlin software (version 6.3, Pharsight, Mountain View, CA, USA). The nominal dose levels and nominal sampling times were used in the calculation of all pharmacokinetic parameters. The linear/log trapezoidal rule was applied in obtaining the PK parameters. NP-G2-044 was observed to be stable after freeze-thaw, and during storage, processing and analysis.

### 4.7. T1 Mammary Tumor Metastasis in Mice

Female BALB/c mice (6- to 8-week old) were purchased from Charles River Labs. The 4T1 tumor cells (1 × 10^5^) were injected subcutaneously into the abdominal mammary gland area of mice using 0.1 mL of a single-cell suspension in PBS-matrigel (*v/v*, 1:1) on Day 0 as previously described [18,19,24]. Starting on Day 7, the NP-G2-044 or control solvent was given once or twice every day by oral gavage at 10, 30, 100 or 300 mg/kg per mouse until Day 27. On Day 28, the mice were sacrificed. This dosage regimen was well tolerated with no signs of overt toxicity. The numbers of metastatic 4T1 cells in lungs were determined by a clonogenic assay. In brief, the lungs were removed from each mouse, finely minced and digested in 5 mL of an enzyme cocktail containing 1 × PBS and 1 mg/mL collagenase type IV for 2 h at 37 ℃ on a platform rocker. After incubation, samples were filtered through 70-µm nylon cell strainers and washed twice with PBS. The resulting cells were suspended and plated with a series of dilutions in 10-cm tissue culture dishes in DMEM medium containing 60 µM thioguanine for clonogenic growth. As 4T1 tumor cells are resistant to 6-thioguanine, the metastasized tumor cells formed foci after 14 days, at which time they were fixed with 4% paraformaldehyde and stained with crystal violet staining solution for counting. For the experiments in Figure 2F,G, 4T1 tumor cells (1 × 10^5^) suspended in PBS-matrigel (*v/v*, 1:1) were injected subcutaneously into the abdominal mammary gland area of mice on day 0. Starting on Day 3, NP-G2-044 was given to mice once every day by oral gavage at 100 mg kg^−1^ per mouse. A vehicle solvent was given to the control group of mice once every day by gavage. Starting on Day 7, paclitaxel was given to mice twice a week by an intraperitoneal injection at 20 mg kg^−1^ per mouse for two weeks. Primary tumors were removed on Day 14. All mice were killed for a clonogenic assay on Day 32.

### 4.8. MDA-MB-231 Human Breast Tumor Metastasis in Mice

MDA-MB-231 cells (subclone LM2) (1 × 10^5^) suspended in PBS-matrigel (*v/v*, 1:1) were injected subcutaneously into the abdominal mammary gland area of female NSG mice (6- to 8-week old from the Jackson Lab) on day 0 as previously described [18,19,24]. Starting on Day 7, NP-G2-044 or the control solvent was given once or twice a day for 6 days every week by gavage until the eighth week. On the first day of the ninth week, the mice were killed. This dosage regimen was well tolerated with no signs of overt toxicity.

The numbers of metastatic MDA-MB-231 cells in lungs were determined by a clonogenic assay. The lungs were removed from each mouse once sacrificed, and the resulting cells were suspended and plated with a series of dilutions in 10-cm tissue culture dishes in medium containing 2.0 µg/mL puromycin for clonogenic growth. As these MBA-MB-231 tumor cells were stably transfected with the vector pSuper-puro, the metastasized tumor cells formed foci after 14 days, at which time they were fixed with 4% paraformaldehyde and stained with crystal violet staining solution for counting. For combination treatments with chemotherapy, MDA-MB-231 tumor cells (1 × 10^5^) suspended in PBS-matrigel (*v/v*, 1:1) were injected subcutaneously into the abdominal mammary gland area of mice on Day 0. Starting on Day 0, 7, or 14, NP-G2-044 was given to mice once a day for 6 days every week by oral gavage at 300 mg/kg per mouse. A vehicle solvent was given to the control mice once a day for 6 days every week. Starting on day 14, doxorubicin hydrochloride (MilliporeSigma, St. Louis, MO, USA) (2 mg/kg) and cyclophosphamide monohydrate (MilliporeSigma) (60 mg/kg) were given to mice once a week for four weeks. Primary tumors were removed on day 28. The death of the mice was used as the endpoint.

### 4.9. Boyden-chamber Cell Migration Assay

As previously described, NCI-H660 cells suspended in 200 μL starvation medium were added to the upper chamber of an insert (6.5 mm diameter, 8 μm pore size; Becton Dickson, Franklin, NJ, USA), and the insert was placed in a 24-well plate containing 400 μL starvation medium with 10% FBS [18,19]. When used, inhibitors were added to both the upper and the lower chambers. Migration assays were performed for 20 h and cells were fixed with 10% paraformaldehyde. Cells were stained with crystal violet staining solution, and cells on the upper side of the insert were removed with a cotton swab. Whole fields on the lower side of the insert were photographed, and the migrated cells were counted. Migration was expressed as the total number of migrated cells.

### 4.10. Mouse Model of Human Neuroendocrine Prostate Cancer

Human neuroendocrine prostate cancer NCI-H660 cells (1 × 10^5^) suspended in PBS were injected into the prostate of male NSG mice (6- to 8-week old from the Jackson Lab) on day 0 as previously described [56]. Briefly, the abdomen was cleaned with iodine solution, and a 1-cm midline incision was created to expose the prostate gland. A 29-gauge needle and a 1-mL disposable syringe were used for the injection of the cell suspension. The needle was inserted into the middle portion of the right anterior lobe, and then was directed towards the cranial portion of the lobe. The abdominal wound was closed in 2 layers with surgical sutures. Starting on Day 7, NP-G2-044 or the control solvent was given once a day for 5 days a week by oral gavage. This dosage regimen was well tolerated with no signs of overt toxicity. The death of the mice was used as the endpoint.

### 4.11. Overall Survival Analysis in Mice

HT cells (5 × 10^6^) suspended in PBS-matrigel (*v/v*, 1: 1) were subcutaneously injected into NSG mice on day 0. Starting on day 28, NP-G2-044 or the control solvent was given once a day for 5 days every week by gavage at 300 mg kg^−1^ per mouse until their death.

For the breast cancer model, MDA-MB-468 cells (5 × 10^6^) suspended in PBS-matrigel (*v/v*, 1:1) were injected subcutaneously into the abdominal mammary gland area of female NSG mice on Day 0 as previously described [18]. Starting on Day 14, NP-G2-044 was given once a day for 5 days every week by gavage at 300 mg kg^−1^ per mouse until their death. Starting on Day 14, gefitinib was given twice weekly by gavage at 150 mg kg^−1^ per mouse until their death. The control solvent was given once a day for 5 days every week by gavage according to their body weight until their death. The primary tumor growth and death of mice were noted for analyzing the tumor growth and survival. The primary tumor volume was calculated as length × width^2^ ×π/6. The primary tumors shown in the figure were dissected on day 55.

For the lymphoma model, U2932 cells (5 × 10^6^) suspended in PBS-matrigel (*v/v*, 1:1) were subcutaneously injected into NSG mice on Say 0. Starting on Day 49, NP-G2-044 was given once a day for 5 days every week by gavage at 300 mg kg^−1^ per mouse until their death. Starting on Day 49, LY2409881 was given once weekly by intraperitoneal injection at 50 mg kg^−1^ per mouse for 8 times. The control solvent was given according to their body weights until their death. The primary tumor growth and death of mice was noted for analyzing tumor growth and survival. The primary tumors and livers shown in the figure were dissected on Day 67.

### 4.12. Statistical Analysis

The median overall survival was analyzed by a log-rank test with the significance defined as *p* < 0.05. For other studies, the statistical significance of differences between groups was evaluated by the paired Student t-test. Data presented are representative of at least three independent experiments. All statistical tests were two-sided, and the results were considered significant at *p* < 0.05.

## 5. Conclusions

Fascin inhibitors can be used to not only inhibit tumor metastasis, but also decrease the tumor growth of specific cancer types.

## Figures and Tables

**Table 1:** Pharmacokinetic (PK) parameters of NP-G2-044 after IV and oral dosing in mice.

Route	Dose (mg/kg)	T_max_ (h)	C_0/max_ (ng/mL)	AUC_(0–t)_ (ng × h/mL)	AUC_(0–inf)_ (ng × h/mL)	T_1__/2_ (h)	MRT_(0–t)_ (h)	MRT_(0–inf)_ (h)	Vd (L/kg)	CL (mL/min/kg)	F (%)
IV	20		11309 ± 3845	7148 ± 1748	7293 ± 1852	5.45 ± 1.58	2.81 ± 0.36	3.34 ± 0.68	9.32 ± 1.35	48.0 ± 13.4	
PO	20	0.83 ± 0.58	541 ± 176	2550 ± 575	2572 ± 568	4.36 ± 0.46	6.16 ± 1.36	6.46 ± 1.44			35.3
PO	50	4.17 ± 3.18	678 ± 258	6186 ± 1648	6214 ± 1649	3.58 ± 0.43	7.40 ± 0.75	7.54 ± 0.85			34.1
PO	100	6.0 ± 3.0	1450 ± 157	14272 ± 2404	14295 ± 2392	4.39 ± 1.56	8.33 ± 0.38	8.40 ± 0.41			39.2

C_max_: The peak plasma concentration of drug after administration. T_max_: Time to reach C_max_. Vd: Volume of distribution (The apparent volume in which a drug is distributed). T_1/2_: Elimination half-life. AUC: Area under the curve (The integral of the concentration-time curve). CL: Clearance (The volume of plasma cleared of the drug per unit time). MRT: mean residence time. F: Bioavailability (The systemically available fraction of a drug) was calculated using AUC_(0–t)_ and nominal dose. IV: intravenous. PO: per oral.

## Supplementary Materials

The following are available online at https://www.mdpi.com/2072-6694/12/8/2287/s1, Figure S1: Full immunoblots from images used in Figure 5A, Figure S2: Full immunoblots from images used in Figure 5B, Figure S3: Full immunoblots from images used in Figure 6E.

## References

[1] Otto J.J., Kane R.E., Bryan J. (1979). Formation of filopodia in coelomocytes: Localization of fascin, a 58,000 dalton actin cross-linking protein. Cell.

[2] Bryan J., Kane R.E. (1978). Separation and interaction of the major components of sea urchin actin gel. J. Mol. Biol..

[3] Yamashiro-Matsumura S., Matsumura F. (1985). Purification and characterization of an F-actin-bundling 55-kilodalton protein from HeLa cells. J. Biol. Chem..

[4] Vignjevic D., Yarar D., Welch M.D., Peloquin J., Svitkina T., Borisy G.G. (2003). Formation of filopodia-like bundles in vitro from a dendritic network. J. Cell Biol..

[5] Vignjevic D., Kojima S., Aratyn Y., Danciu O., Svitkina T., Borisy G.G. (2006). Role of fascin in filopodial protrusion. J. Cell Biol..

[6] Darnel A.D., Behmoaram E., Vollmer R.T., Corcos J., Bijian K., Sircar K., Su J., Jiao J., Alaoui-Jamali M.A., Bismar T.A. (2009). Fascin regulates prostate cancer cell invasion and is associated with metastasis and biochemical failure in prostate cancer. Clin. Cancer Res..

[7] Pelosi G., Pasini F., Fraggetta F., Pastorino U., Iannucci A., Maisonneuve P., Arrigoni G., De Manzoni G., Bresaola E., Viale G. (2003). Independent value of fascin immunoreactivity for predicting lymph node metastases in typical and atypical pulmonary carcinoids. Lung Cancer.

[8] Hashimoto Y., Shimada Y., Kawamura J., Yamasaki S., Imamura M. (2004). The prognostic relevance of fascin expression in human gastric carcinoma. Oncology.

[9] Cao D., Ji H., Ronnett B.M. (2005). Expression of mesothelin, fascin, and prostate stem cell antigen in primary ovarian mucinous tumors and their utility in differentiating primary ovarian mucinous tumors from metastatic pancreatic mucinous carcinomas in the ovary. Int. J. Gynecol. Pathol..

[10] Rodriguez-Pinilla S.M., Sarrio D., Honrado E., Hardisson D., Calero F., Benitez J., Palacios J. (2006). Prognostic significance of basal-like phenotype and fascin expression in node-negative invasive breast carcinomas. Clin. Cancer Res..

[11] Machesky L.M., Li A. (2010). Fascin: Invasive filopodia promoting metastasis. Commun. Integr. Biol..

[12] Tan V.Y., Lewis S.J., Adams J.C., Martin R.M. (2013). Association of fascin-1 with mortality, disease progression and metastasis in carcinomas: A systematic review and meta-analysis. BMC Med..

[13] Grothey A., Hashizume R., Sahin A.A., McCrea P.D. (2000). Fascin, an actin-bundling protein associated with cell motility, is upregulated in hormone receptor negative breast cancer. Br. J. Cancer.

[14] Hashimoto Y., Skacel M., Adams J.C. (2005). Roles of fascin in human carcinoma motility and signaling: Prospects for a novel biomarker?. Int. J. Biochem. Cell Biol..

[15] Yamakita Y., Matsumura F., Yamashiro S. (2009). Fascin1 is dispensable for mouse development but is favorable for neonatal survival. Cell Motil. Cytoskeleton.

[16] Li A., Morton J.P., Ma Y., Karim S.A., Zhou Y., Faller W.J., Woodham E.F., Morris H.T., Stevenson R.P., Juin A. (2014). Fascin is regulated by slug, promotes progression of pancreatic cancer in mice, and is associated with patient outcomes. Gastroenterology.

[17] Schoumacher M., El-Marjou F., Lae M., Kambou N., Louvard D., Robine S., Vignjevic D.M. (2014). Conditional expression of fascin increases tumor progression in a mouse model of intestinal cancer. Eur. J. Cell Biol..

[18] Huang F.K., Han S., Xing B., Huang J., Liu B., Bordeleau F., Reinhart-King C.A., Zhang J.J., Huang X.Y. (2015). Targeted inhibition of fascin function blocks tumour invasion and metastatic colonization. Nat. Commun..

[19] Han S., Huang J., Liu B., Xing B., Bordeleau F., Reinhart-King C.A., Li W., Zhang J.J., Huang X.Y. (2016). Improving fascin inhibitors to block tumor cell migration and metastasis. Mol. Oncol..

[20] Huang J., Dey R., Wang Y., Jakoncic J., Kurinov I., Huang X.Y. (2018). Structural Insights into the Induced-fit Inhibition of Fascin by a Small-Molecule Inhibitor. J. Mol. Biol..

[21] Davies B., Morris T. (1993). Physiological parameters in laboratory animals and humans. Pharm. Res..

[22] Shan D., Chen L., Njardarson J.T., Gaul C., Ma X., Danishefsky S.J., Huang X.Y. (2005). Synthetic analogues of migrastatin that inhibit mammary tumor metastasis in mice. Proc. Natl. Acad. Sci. USA.

[23] Yang S., Zhang J.J., Huang X.Y. (2009). Orai1 and STIM1 are critical for breast tumor cell migration and metastasis. Cancer Cell.

[24] Chen L., Yang S., Jakoncic J., Zhang J.J., Huang X.Y. (2010). Migrastatin analogues target fascin to block tumour metastasis. Nature.

[25] Pulaski B.A., Ostrand-Rosenberg S. (1998). Reduction of established spontaneous mammary carcinoma metastases following immunotherapy with major histocompatibility complex class II and B7.1 cell-based tumor vaccines. Cancer Res..

[26] Pinkus G.S., Pinkus J.L., Langhoff E., Matsumura F., Yamashiro S., Mosialos G., Said J.W. (1997). Fascin, a sensitive new marker for Reed-Sternberg cells of hodgkin’s disease. Evidence for a dendritic or B cell derivation?. Am. J. Pathol..

[27] Kloo B., Nagel D., Pfeifer M., Grau M., Duwel M., Vincendeau M., Dorken B., Lenz P., Lenz G., Krappmann D. (2011). Critical role of PI3K signaling for NF-kappaB-dependent survival in a subset of activated B-cell-like diffuse large B-cell lymphoma cells. Proc. Natl. Acad. Sci. USA.

[28] Deng C., Lipstein M., Rodriguez R., Serrano X.O., McIntosh C., Tsai W.Y., Wasmuth A.S., Jaken S., O’Connor O.A. (2015). The novel IKK2 inhibitor LY2409881 potently synergizes with histone deacetylase inhibitors in preclinical models of lymphoma through the downregulation of NF-kappaB. Clin. Cancer Res..

[29] Lawrence R.T., Perez E.M., Hernandez D., Miller C.P., Haas K.M., Irie H.Y., Lee S.I., Blau C.A., Villen J. (2015). The proteomic landscape of triple-negative breast cancer. Cell Rep..

[30] Rijken P.J., Hage W.J., van Bergen en Henegouwen P.M., Verkleij A.J., Boonstra J. (1991). Epidermal growth factor induces rapid reorganization of the actin microfilament system in human A431 cells. J. Cell Sci..

[31] Van Bergen en Henegouwen P.M., den Hartigh J.C., Romeyn P., Verkleij A.J., Boonstra J. (1992). The epidermal growth factor receptor is associated with actin filaments. Exp. Cell Res..

[32] Den Hartigh J.C., van Bergen en Henegouwen P.M., Verkleij A.J., Boonstra J. (1992). The EGF receptor is an actin-binding protein. J. Cell Biol..

[33] Weiss L. (2000). Metastasis of cancer: A conceptual history from antiquity to the 1990s. Cancer Metastasis Rev..

[34] Fidler I.J. (2003). The pathogenesis of cancer metastasis: The ‘seed and soil’ hypothesis revisited. Nat. Rev. Cancer.

[35] Valastyan S., Weinberg R.A. (2011). Tumor metastasis: Molecular insights and evolving paradigms. Cell.

[36] Condeelis J., Singer R.H., Segall J.E. (2005). The great escape: When cancer cells hijack the genes for chemotaxis and motility. Annu. Rev. Cell Dev. Biol..

[37] Pollard T.D., Cooper J.A. (2009). Actin, a central player in cell shape and movement. Science.

[38] Mattila P.K., Lappalainen P. (2008). Filopodia: Molecular architecture and cellular functions. Nat. Rev. Mol. Cell Biol..

[39] Coopman P.J., Do M.T., Thompson E.W., Mueller S.C. (1998). Phagocytosis of cross-linked gelatin matrix by human breast carcinoma cells correlates with their invasive capacity. Clin. Cancer Res..

[40] Mogilner A., Rubinstein B. (2005). The physics of filopodial protrusion. Biophys. J..

[41] Yao H., He G., Yan S., Chen C., Song L., Rosol T.J., Deng X. (2017). Triple-negative breast cancer: Is there a treatment on the horizon?. Oncotarget.

[42] Vlachostergios P.J., Puca L., Beltran H. (2017). Emerging Variants of Castration-Resistant Prostate Cancer. Curr. Oncol. Rep..

[43] Rickman D.S., Beltran H., Demichelis F., Rubin M.A. (2017). Biology and evolution of poorly differentiated neuroendocrine tumors. Nat. Med..

[44] Al-Alwan M., Olabi S., Ghebeh H., Barhoush E., Tulbah A., Al-Tweigeri T., Ajarim D., Adra C. (2011). Fascin is a key regulator of breast cancer invasion that acts via the modification of metastasis-associated molecules. PLoS ONE.

[45] Groen C.M., Jayo A., Parsons M., Tootle T.L. (2015). Prostaglandins regulate nuclear localization of Fascin and its function in nucleolar architecture. Mol. Biol. Cell.

[46] Jayo A., Malboubi M., Antoku S., Chang W., Ortiz-Zapater E., Groen C., Pfisterer K., Tootle T., Charras G., Gundersen G.G. (2016). Fascin Regulates Nuclear Movement and Deformation in Migrating Cells. Dev. Cell.

[47] Chiasson-MacKenzie C., McClatchey A.I. (2018). EGFR-induced cytoskeletal changes drive complex cell behaviors: The tip of the iceberg. Sci. Signal..

[48] Roth L., Srivastava S., Lindzen M., Sas-Chen A., Sheffer M., Lauriola M., Enuka Y., Noronha A., Mancini M., Lavi S. (2018). SILAC identifies LAD1 as a filamin-binding regulator of actin dynamics in response to EGF and a marker of aggressive breast tumors. Sci. Signal..

[49] Pike R., Ortiz-Zapater E., Lumicisi B., Santis G., Parsons M. (2018). KIF22 coordinates CAR and EGFR dynamics to promote cancer cell proliferation. Sci. Signal..

[50] Moujaber O., Stochaj U. (2020). The Cytoskeleton as Regulator of Cell Signaling Pathways. Trends Biochem. Sci..

[51] Hu H., Juvekar A., Lyssiotis C.A., Lien E.C., Albeck J.G., Oh D., Varma G., Hung Y.P., Ullas S., Lauring J. (2016). Phosphoinositide 3-Kinase Regulates Glycolysis through Mobilization of Aldolase from the Actin Cytoskeleton. Cell.

[52] Arnold H., Pette D. (1970). Binding of aldolase and triosephosphate dehydrogenase to F-actin and modification of catalytic properties of aldolase. Eur. J. Biochem..

[53] Wang J., Morris A.J., Tolan D.R., Pagliaro L. (1996). The molecular nature of the F-actin binding activity of aldolase revealed with site-directed mutants. J. Biol. Chem..

[54] Wang Y., Lei R., Zhuang X., Zhang N., Pan H., Li G., Hu J., Pan X., Tao Q., Fu D. (2014). DLC1-dependent parathyroid hormone-like hormone inhibition suppresses breast cancer bone metastasis. J. Clin. Investig..

[55] Nabbi A., Riabowol K. (2015). Rapid Isolation of Nuclei from Cells In Vitro. Cold Spring Harb. Protoc..

[56] Cifuentes F.F., Valenzuela R.H., Contreras H.R., Castellon E.A. (2015). Development of an orthotopic model of human metastatic prostate cancer in the NOD-SCIDgamma mouse (Mus musculus) anterior prostate. Oncol. Lett..

