# Supplementary material for: Anti-Metastasis Fascin Inhibitors Decrease the Growth of Specific Subtypes of Cancers
Source: Cancers (Basel). 2020 Aug 14;12(8):2287. doi: 10.3390/cancers12082287 (PMC7466159; doi:10.3390/cancers12082287)
Supplement: Supplementary file 1 [file cancers-12-02287-s001.pdf]

cancers

MDPI

# Supplementary Materials: Anti-Metastasis Fascin Inhibitors Decrease the Growth of Specific Subtypes of Cancers

**Yufeng Wang, J. Jillian Zhang and Xin-Yun Huang**

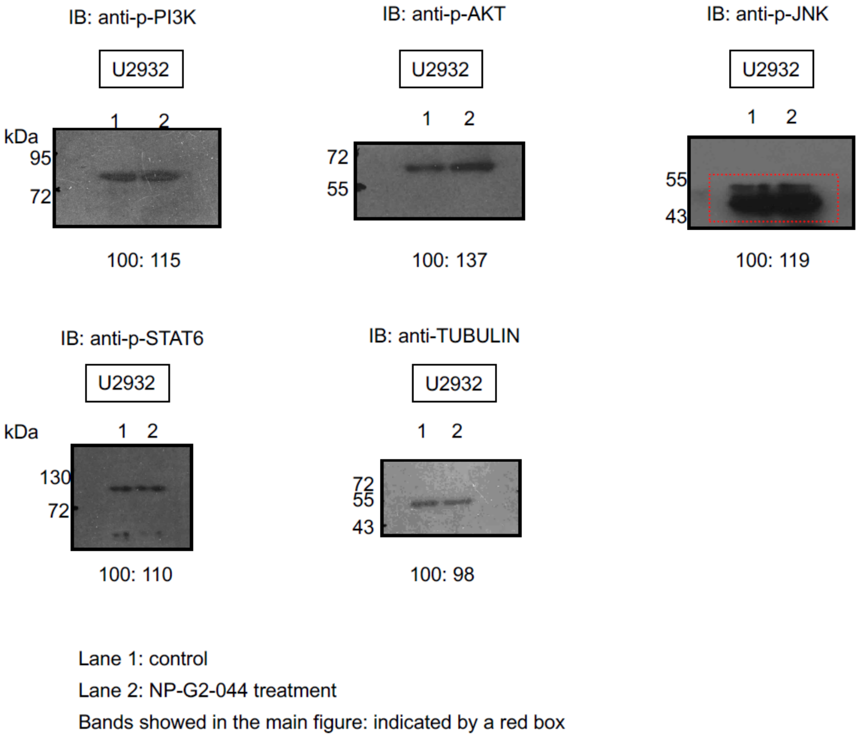


**Figure S1.** Immunoblots form images used in Figure 5A.

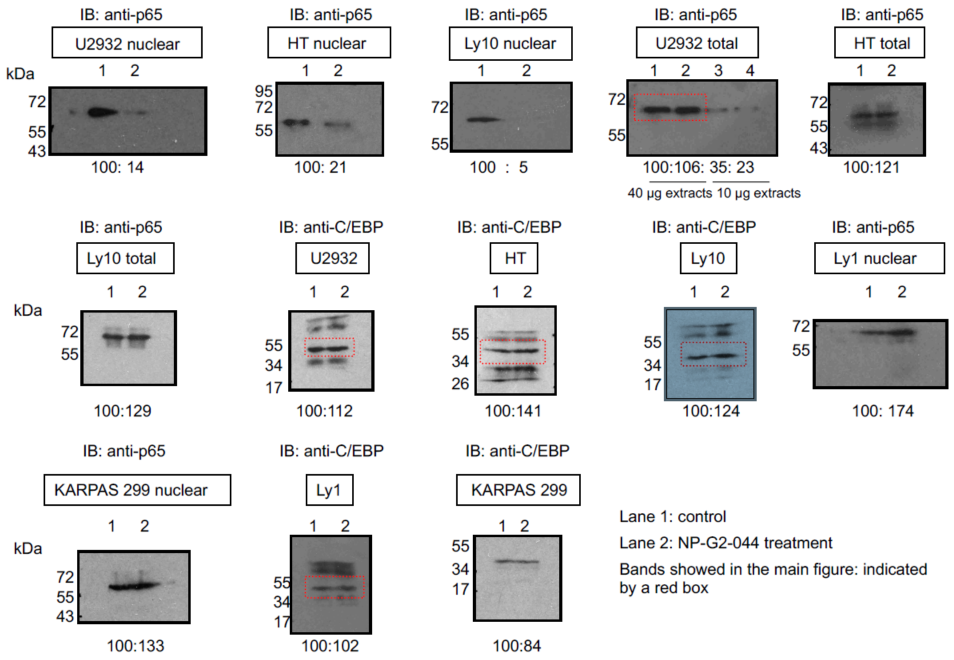


**Figure S2.** Immunoblots from image used in Figure 5B.

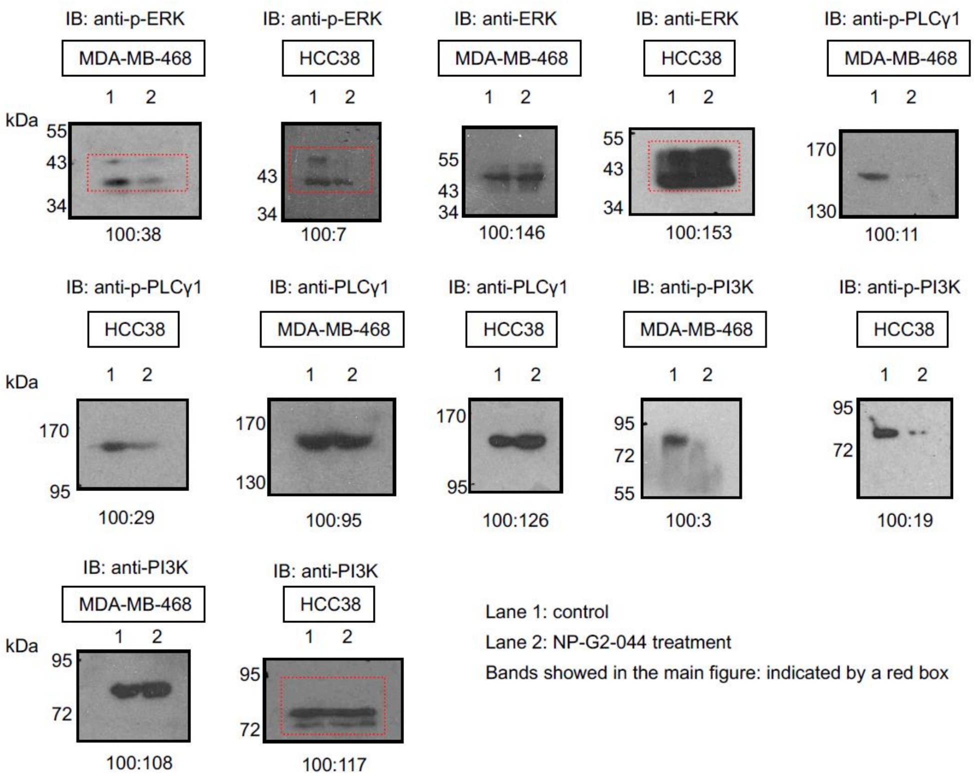


**Figure S3.** Immunoblots from image used in Figure 6E.

© 2020 by the authors. Licensee MDPI, Basel, Switzerland. This article is an open access article distributed under the terms and conditions of the Creative Commons Attribution (CC BY) license (http://creativecommons.org/licenses/by/4.0/).
